# Conditional deletion of *Ahr* alters gene expression profiles in hematopoietic stem cells

**DOI:** 10.1371/journal.pone.0206407

**Published:** 2018-11-02

**Authors:** John A. Bennett, Kameshwar P. Singh, Stephen L. Welle, Lisbeth A. Boule, B. Paige Lawrence, Thomas A. Gasiewicz

**Affiliations:** 1 Department of Environmental Medicine, University of Rochester Medical Center, Rochester, New York, United States of America; 2 Department of Medicine, University of Rochester Medical Center, Rochester, New York, United States of America; St. Vincent's Institute, AUSTRALIA

## Abstract

The aryl hydrocarbon receptor (AHR) is a ligand activated bHLH transcription factor that belongs to the Per-Arnt-Sim (PAS) superfamily of proteins involved in mediating responses to cellular environment regulating normal physiological and developmental pathways. The AHR binds a broad range of naturally derived and synthetic compounds, and plays a major role in mediating effects of certain environmental chemicals. Although our understanding of the physiological roles of the AHR in the immune system is evolving, there is little known about its role in hematopoiesis and hematopoietic diseases. Prior studies demonstrated that AHR null (AHR-KO) mice have impaired hematopoietic stem cell (HSC) function; they develop myeloproliferative changes in peripheral blood cells, and alterations in hematopoietic stem and progenitor cell populations in the bone marrow. We hypothesized mice lacking AHR expression only within hematopoietic cells (AHR^Vav1^ mice) would develop similar changes. However, we did not observe a complete phenocopy of AHR-KO and AHR^Vav1^ animals at 2 or 18 months of age. To illuminate the signaling mechanisms underlying the alterations in hematopoiesis observed in these mice, we sorted a population of cells highly enriched for HSC function (LSK cells: CD34-CD48-CD150+) and performed microarray analyses. Ingenuity Pathway and Gene Set Enrichment Analyses revealed that that loss of AHR within HSCs alters several gene and signaling networks important for HSC function. Differences in gene expression networks among HSCs from AHR-KO and AHR^Vav1^ mice suggest that AHR in bone marrow stromal cells also contributes to HSC function. In addition, numerous studies have suggested a role for AHR in both regulation of hematopoietic cells, and in the development of blood diseases. More work is needed to define what these signals are, and how they act upon HSCs.

## Introduction

All mature lineages of blood cells are generated from hematopoietic stem cells (HSCs), which reside primarily in bone marrow (BM) of adult mice and humans. One of the most important aspects of HSC biology is the precise regulation of their proliferation, differentiation, and self-renewal. This balance can be shifted due to genetic mutations, environmental exposures to toxicants, and age [1–5]. For example, exposure to environmental toxicants which activate the aryl hydrocarbon receptor (AHR) have been linked to blood diseases in humans.

The aryl hydrocarbon receptor (AHR) is an environment sensing transcriptional regulator that is expressed in hematopoietic and non-hematopoietic cells. While the normal, physiological role of AHR is not fully understood, it regulates aspects of HSC function, immune system development, and hematopoietic diseases [3, 6–11]. Several proposed physiological functions of AHR in non-hematopoietic tissues have been suggested from studies using AHR-null-allele (AHR-KO) mouse models [9, 12, 13]. We have summarized these previous data in Table 1. While these models have generated much information on possible roles of the receptor in a variety of tissues and cell types, few studies have sought to describe the role of AHR as an intrinsic regulator of BM stem cell functions. Hematopoietic cells, including HSCs, exist in the BM in close proximity to a variety of other cell types. Multiple studies that have described the role of these non-hematopoietic cells in the regulation of HSC function have led to the development of models that describe a hematopoietic “niche”, the cells of which can have significant regulatory effects on HSCs and greatly alter their function and output [14–19].

**Table 1:** Summary of phenotypes observed in global AHR-KO mice.

Phenotypes Observed in Global AHR-KO mice
Increased numbers of peripheral white blood cells
Alterations in white blood cell subsets
Elevated HSC oxidative stress elevated
HSC DNA damage increased
HSC p16 expression decreased
Spleen weight increased
Decreased HSC self-renewal during serial transplants
672 genes altered compared to WT using microarray

In order to better understand the role of AHR signaling intrinsic to HSCs, we used a conditional knockout (AHR^Vav1^) model that utilizes a Cre-loxP system to target loss of AHR expression to the hematopoietic system [20, 21]. Targeted deletion of AHR allows for better discrimination of genes that are regulated intrinsically, and avoids the potential confounding effects in global knockout because all cells, including BM stromal cells, lack AHR expression. Here we report the characterization of the morphology and hematopoietic phenotype of AHR^Vav1^ mice, as well as the gene expression profiles of long-term HSC (LT-HSC) from these mice at 8 weeks or 18 months of age. LT-HSCs are the most primitive multipotent BM stem cell, and are responsible for long term multi-lineage cell generation. These data provide insight into the genes that AHR may intrinsically regulate within HSCs, and guide new approaches to elucidate how modulation or loss of AHR signaling can lead to diseases or dysfunction of the hematopoietic system.

## Materials and methods

### Mice

Breeding pairs of B6.Cg-Tg A2Kio/J (Vav1-Cre) and *AHR*^*tm3*.*1Bra*^/J (AHR-KO) mice were obtained from Jackson Laboratories (Bar Harbor, Maine). Initial breeding stocks for Ahr^fx/fx^mice were provided by Dr. Christopher Bradfield (University of Wisconsin) [20]. All mice were maintained in micro-isolator housing according to approved protocols at the University of Rochester. Males expressing Vav1-Cre were crossed with females homozygous for the floxed *Ahr* allele. This breeding scheme produced males heterozygous for both transgenes, which were then crossed with female *Ahr*^*f*x/fx^ mice, to produce female *Vav1-cre*^+/-^*Ahr*^fx/fx^ (AHR^Vav1^) mice. These female mice were used in the experiments presented. Female *Ahr*^fx/fx^ (AHR^FX^) mice were used as experimental controls. The CD45.1^+^ (B6.SJL-Ptprc<a>/BoAiTac) mice used in the repopulation experiments were purchased from Taconic Farms (Rensselaer, New York).

### Ethics statement

Animals were used at 2 or 18 months of age. All handling and experimental procedures were carried out in accordance with University of Rochester approved protocols. Our study was approved by both the University Committee on Animal Resources (UCAR) as well as the Institutional Biosafety Committee (IBC). Mice are housed in cages with air-handling systems designed to reduce ammonia build-up. Food and water are changed frequently by dedicated animal care technicians and were provided ab libitum for all studies described here. Mice receive nesting materials and red plastic houses to minimize distress and to encourage natural behaviors such as nesting and familial socialization. Prior to experiments, all mice were sacrificed using CO_2_ asphyxiation and cervical dislocation to ensure death and absence of pain due to tissue harvests. Mice are monitored daily by dedicated vivarium staff for health and well-being. These checks monitor appearance, behavior and food and water levels to ensure the health and comfort of all experimental animals.

### Cell isolation and detection of Ahr excision by PCR

Cells were isolated from the bone marrow, spleen, lymph nodes, and lung as previously described [11, 22, 23]. After hypotonic lysis to remove erythrocytes, single cell suspensions were prepared. CD45^negative^ (non-hematopoietic cells) were isolated from the lung. To do so, 1% low melting point agarose was inserted into the lung via the trachea, and the tissue was then digested with dispase. CD45^negative^ cells were isolated from the resulting cell suspension using a magnetic enrichment beads conjugated to an anti-mouse CD45 antibody (Miltenyi Biotec, Auburn, CA). Genomic DNA was isolated from cell suspensions using a QIAamp DNA Mini Kit (QIAGEN), and PCR to detect the excised *Ahr* allele was performed as previously described [20, 23, 24].

### Western Blotting for AHR protein in hematopoietic tissues

Cells from AHR^Vav1^ tissues were washed twice with PBS, and lysed (Reporter Lysis Buffer, Promega, Madison, WI). Lysates were stored at −80°C. Lysate proteins were separated by SDS-polyacrylamide gel electrophoresis (PAGE) (8% acrylamide resolving gel) and transferred to a polyvinylidene fluoride (PVDF) membrane, which was blocked using 5% nonfat milk in wash buffer (50mM Tris base, 150mM NaCl, and 0.2% Tween 20, pH 7.5). Antibodies used were anti-AHR (rabbit polyclonal, Enzo Life Sciences, Farmingdale NY) and anti-β-actin (rabbit polyclonal, Sigma, St Louis, MO). Horseradish peroxidase-conjugated secondary antibodies were purchased from Jackson Immunoresearch (West Grove, PA). Proteins were visualized by chemiluminescence using LumiGlo reagents (KPL, Gaithersburg, MD).

### Tissue and cell collection and hematological profiles

Mice were euthanized with CO_2_ and peripheral blood was collected from the retroorbital plexus vein into BD microtainer tubes containing EDTA (Beckton Dickinson and Company). Hematological profiles were analyzed using a HESKA Hematology Analyzer (HESKA Corporation, Colorado). Organs were harvested to collect wet weights. BM cells from both femurs of each mouse were harvested as previously described [6]. The number of cells obtained was determined using a hemocytometer.

### Bone marrow cell phenotyping

A single cell suspension of BM cells was prepared and stained with a cocktail of fluorochrome-conjugated antibodies against lineage markers defining more mature hematopoietic cells as previously described [6]. Lineage depleted cell suspensions from BM were stained with fluorochrome-conjugated antibodies to detect the following populations of cells: long-term repopulating HSCs (LT-HSCs) (Lin^−^CD34^-^Flt3^-^Sca-1^high^cKit^+^), short-term HSCs (ST-HSCs) (Lin-CD34^+^Flt3^-^Sca-1^high^cKit^+^), MPP (Lin^-^Sca-1^+^cKit^high^Flt3^+^), common lymphoid progenitors (CLP) (Lin-IL7Rα^+^Sca-1^low^cKit^+^), common myeloid progenitors (CMP) (Lin^−^CD34^+^FcR^low^IL7Rα^−^Sca-1^−^cKit^+^), granulocyte macrophage progenitors (GMP) (Lin^−^CD34^+^FcR^high^IL7Rα^−^Sca-1^−^cKit^+^), and megakaryocyte erythroid progenitors (MEP) (Lin^−^CD34^−^FcR^low^IL7Rα^−^Sca-1^−^cKit^+^). The antibody clones used to analyze BM cells were CD34 (RAM34), Sca-1 (E13-161.7), cKit (2B8), IL7R (A7R34), and FcR (2.4G2) (BD Pharmingen). Spleen cells were analyzed using fluorochrome-conjugated antibodies against CD4 (H129.19), CD8 (53–6.7), B220 (RA3-6B2), Gr-1(RB6-8C5), and Mac-1(M1/70) (BD Pharmingen). Bone marrow cells (Lin-) were also stained with CD48 (FITC clone Hm48-1; BD Pharmingen) and CD150 (APC Clone 459911; R&D Systems) to identify LT-HSCs for cell sorting. When indicated, cells were also stained with 10 μM 2’7’-dichlorofluorescein diacetate (DCFDA) (Abcam) to measure cellular oxidative status [7]. All flow cytometry was performed on a Becton Dickenson LSRII flow cytometer maintained by the University of Rochester Flow Cytometry Core. Flow data were analyzed using Flowjo software (Treestar, California).

### Spleen colony forming unit assay

Recipient C57/Bl6 mice were sub-lethally irradiated (9.0 Gy) using a ^137^Cs gamma-ray source and after 4 h these mice were tail-vein injected with 50,000 total BM cells from donor AHR^FX^ or AHR^Vav1^ (prepared as described above) suspended in PBS. The recipient mice were euthanized after 8 or 12 days. Spleens were collected and fixed in Telleyesniczky’s solution. The nodular colonies formed on the spleens were counted macroscopically [2].

### Serial Bone marrow transplant assay

Bone marrow cells from recipient (CD45.1^+^) and donor AHR^Vav1^ or AHR^FX^ (CD45.2^+^) mice were prepared as described earlier [7]. Cells isolated from AHR^Vav1^ or AHR^FX^ (1 x 10^6^) and CD45.1^+^ (1 x 10^6^) mice were injected together (in 100 μl PBS) into the tail vein of irradiated (550 + 550 rads, 4 h apart) CD45.1^+^ recipients. After 20 weeks, BM cells from recipient animals were harvested and injected (2 x 10^6^ cells) into irradiated CD45.1^+^ secondary recipient mice. Similarly, BM cells isolated from secondary recipients and injected into tertiary recipients. Bone marrow cells were isolated from primary, secondary, and tertiary CD45.1^+^ recipients were analyzed for cells of donor (CD45.2^+^) and recipient (CD45.1^+^) origins. Bone marrow engraftment was analyzed at 20 weeks after each BM transplantation.

### Sorting and microarray analysis of Lin-CD48-CD150+ (SLAM+) cells

BM cells from femurs and tibias were harvested from each animal (6 to 8 mice) separately and depleted for lineage positive cells as previously described [6]. The Lin- cells were stained with fluorochrome-conjugated antibodies against Sca-1 (V450 Clone D7; BD Pharmingen), cKit (PeCy7 Clone 2B8; BD Pharmingen), CD34 (AF700 Clone Ram34; eBiosciences), CD48 (FITC Clone Hm48-1; BD Pharmingen), and CD150 (APC Clone 459911; R&D Systems). HSC were obtained by fluorescence-activated cell sorting lineage depleted BM cells [7]. Cells were sorted into RLT+ buffer (Qiagen) and placed at -80°C. Total RNA was isolated from sorted LT-HSCs using an RNeasy Mini Kit (Qiagen). RNA was pre-amplified and cDNA was produced using a WT-Ovation PicoSL kit (Nugen). Each animal was ultimately prepared as a cDNA library for analysis using a distinct microarray chip. Microarray analysis was performed using Genechip Mouse Gene 2.0 ST Array (Affymetrix) by the UR Genomics Research Center. The IterPLIER algorithm was used to generate background-subtracted, quantile-normalized signals from the microarray data. Transcripts with a mean difference in expression of at least 1.5-fold and P<0.05 were examined for network/pathway associations by Ingenuity Pathway Analysis. Physiological functions were ascribed to the altered set of genes. Microarray data were also analyzed using Gene Set Enrichment Analysis (GSEA) as previously described [7]. The microarray data can be accessed through the Gene Expression Omnibus accession number GSE76276.

### Reverse transcription (RT)-qPCR

LT-HSCs (SLAM+) were sorted as described above, and total RNA was extracted using an RNeasy Mini Kit (Qiagen). cDNA was prepared as described above and 10 ng of cDNA was used in qPCR reactions via a Bio-Rad CFX96 Real Time Thermal Cycler (Bio-Rad, Hercules, California), using primer/probe assays (Applied Biosystems). Expression of mRNA for each gene was normalized using the expression of *Hprt* and *Gapdh*. Gene expression was compared using the 2^-ΔΔT^ method [25]. Differences were considered significant when relative mRNA expression was 1.5-fold higher or lower than indicated controls with a p-value of less than 0.05.

### Statistics

For analyses of two groups, the Student’s t-test was used. For comparisons of two or more groups, a two-way ANOVA was used. For microarray data, The IterPLIER algorithm was used to generate background-subtracted, quantile-normalized signals from the microarray data and analyses were adjusted to account for multiple comparisons. Ingenuity Pathways Analysis (IPA) uses a proprietary platform knowledge base and Fischer’s Exact Test to determine if pathways and subsets of genes are altered.

## Results

### Validation of conditional deletion of *Ahr* gene in hematopoietic cells

**Fig 1:**
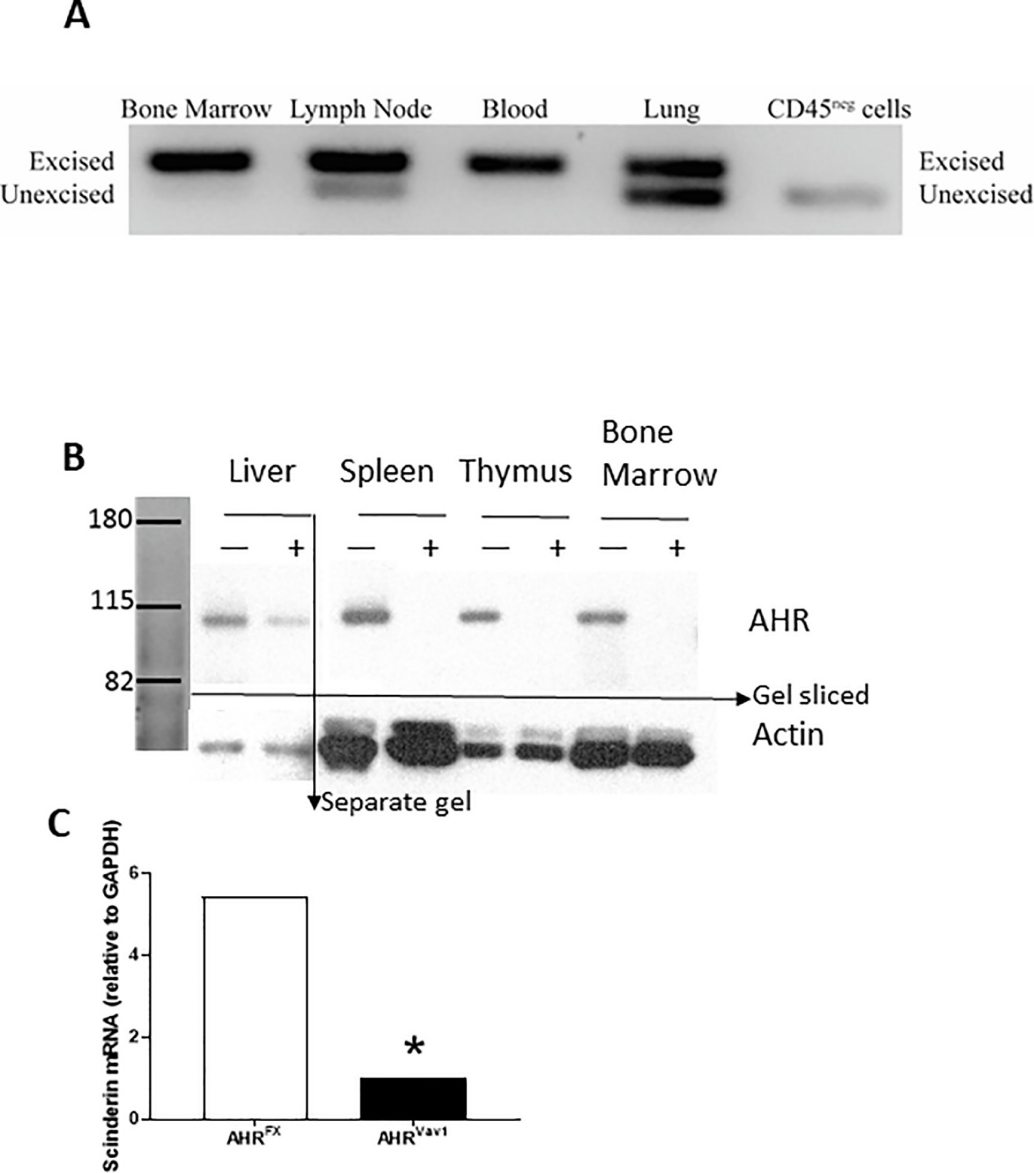
Genotyping and gene induction in 8 week old AHR^Vav1^ mice. (A) Excised *Ahr* is detectable in hematopoietic tissues of AHR^Vav1^ animals. Tissues from AHR^Vav1^ and AHR^FX^ mice were isolated as described and DNA was extracted for PCR analysis using a three primer reaction that amplifies either 180 bp fragments (excised allele) or 140 bp (unexcised allele) fragments. (B) AHR^Vav1^ animals have reduced levels of AHR protein in hematopoietic cells. Various tissues (liver, spleen, thymus, and total bone marrow) were analyzed by western blot to detect AHR (~90kD). AHR^FX^ mice (+) express the receptor in all tissues. AHR^Vav1^ (-) mice display reductions or lack of AHR in the tissues examined. Hepatoma cells were used as a positive control as they express large amounts of AHR.(C) Lineage negative cells from AHR^Vav1^ mice fail to upregulate Scinderin mRNA after 6h exposure to 10nM TCDD. Data expressed relative to DMSO vehicle, using HPRT as an endogenous control gene for normalization. N = 4 mice per treatment group. Relative fold change in expression was calculated using the 2^ΔΔCt^ method.

To verify that targeted excision is occurring, we isolated genomic DNA from spleen, liver, kidney, thymus and BM from AHR^FX^ and AHR^Vav1^ animals. PCR analyses revealed that hematopoietic tissues from AHR^Vav1^ mice display excision of *Ahr* (Fig 1A). The tissues analyzed included the hematopoietic-derived cell enriched tissues BM, spleen, thymus. We determined that CD45 non-hematopoietic cells only display the unexcised allele. Western blotting confirmed that hematopoietic tissues from AHR^Vav1^ mice have greatly reduced or undetectable AHR protein (Fig 1B). As an additional demonstration of functional loss of AHR, we treated isolated BM cells with the potent AHR agonist TCDD, and measured the expression level of the TCDD:AHR gene target *Scinderin* [3] by RT-qPCR. Relative to isolated Lin- BM cells from TCDD treated with control, *Scinderin* expression is 5-fold higher in Lin- BM cells from AHR^FX^ mice exposed to TCDD ex vivo (Fig 1C). In contrast, Lin- BM cells from AHR^Vav1^ mice failed to respond to TCDD, which further supports that functional deletion of AHR protein was achieved (Fig 1C). Some reports have suggested that *Ahr* excision is possible in non-hematopoietic tissues due to endothelial expression of Vav1-cre [26], but we did not observe this in our PCR screens. Combined with our confirmation that AHR protein levels are reduced in hematopoietic tissues in AHR^Vav1^ mice, and that they do not respond to TCDD, AHR^Vav1^ mice are a useful model for studying the hematopoietic role of AhR. Our data shows that we have deleted AhR within hematopoietic cells, and not other tissues.

Flanking *Ahr* with loxp sites and/or the expression of the *Cre* transgene could potentially have unintended off-target effects. AHR^Vav1^ animals were healthy and did not display any gross alterations to non-hematopoietic organs (S1 Fig). No difference was observed in total body mass, indicating that the mice grow at the same rate as controls. Heart, lung, thymus, kidney, liver and spleen mass were unaltered in AHR^Vav1^ mice relative to age-matched AHR^FX^ controls. Together these data indicate that neither deletion of the *Ahr* gene in hematopoietic tissues nor expression of the *Cre* transgene is sufficient to induce observable gross changes in non-hematopoietic organs.

**Fig 2:**
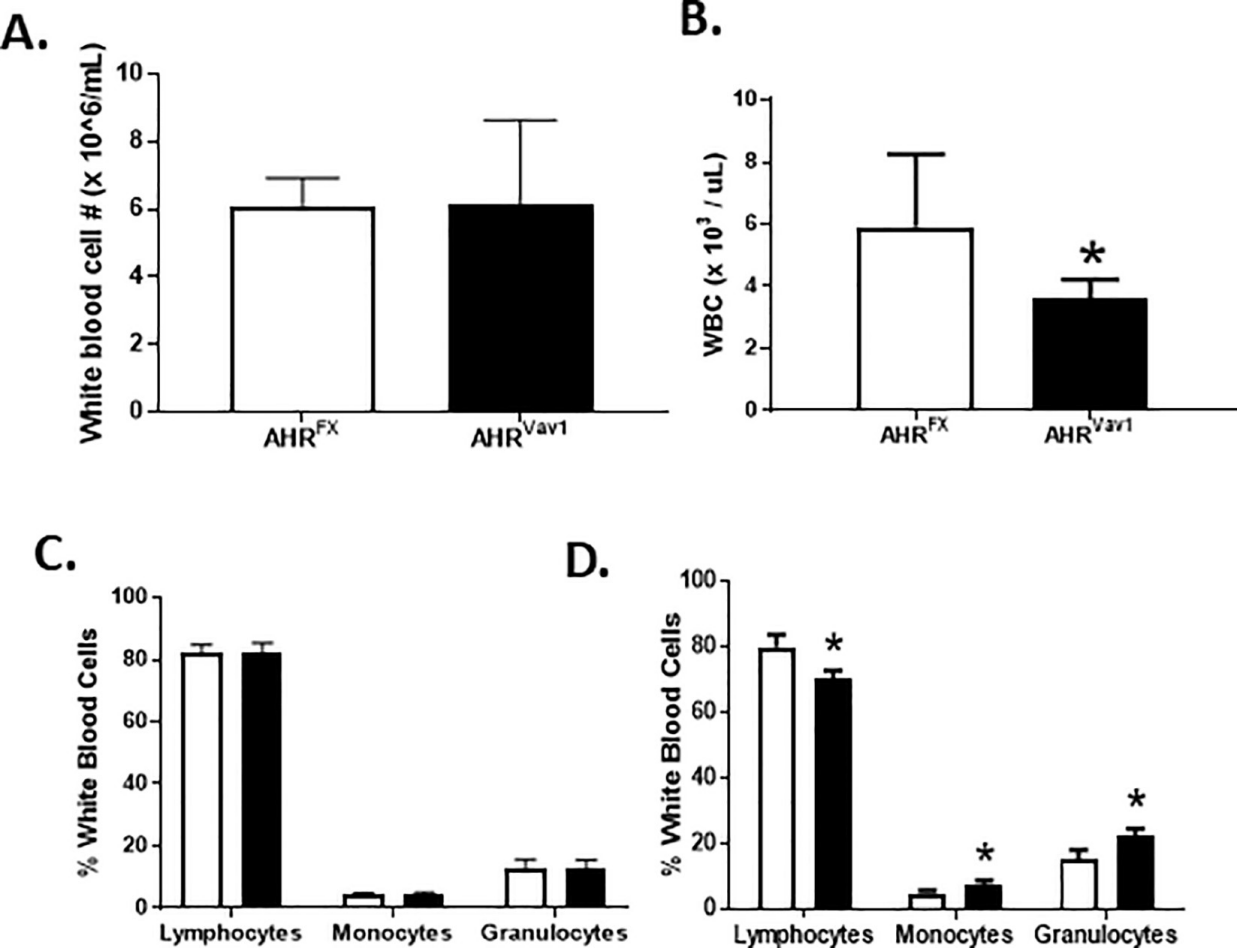
Aging AHR^Vav1^ mice show changes in total and differential WBC counts. Peripheral blood was collected from retro-orbital plexus and complete blood cell counts were done using HESKA blood cell counter: 8-week-old (A & C), 18-month-old (B & D). * = p<0.05 compared to AHR^FX^ controls. N = 5 mice per group.

Alterations to peripheral blood cells can be reflective of defects in HSC function. AHR-KO mice display alterations to peripheral blood composition at 2- and 24-months [6, 7]. To determine if lack of AHR in hematopoietic cells is sufficient to produce similar alterations in peripheral blood, we examined complete blood counts and hematological parameters in 2- and 18-month old AHR^Vav1^ mice compared to age-matched AHR^FX^ mice. Young AHR^Vav1^ mice displayed no alterations to the total white blood cell count or in the percentage of lymphocytes, monocyte or granulocyte in peripheral blood (Fig 2A and 2C). However, as these mice age, there was significant decrease in total white blood cell (WBC) number. In addition, there was significant increase in monocytes and granulocytes, but a decrease in lymphocytes in older AHR^Vav1^ mice (Fig 2B and 2D).However, spleens from 8-week old AHR^Vav1^ mice did not display alterations in cellularity compared to AHR^FX^ controls, nor were there significant differences in the percentage or number of T-cells, B-cells, myeloid cells and erythroid cells (S2 Fig).

**Fig 3:**
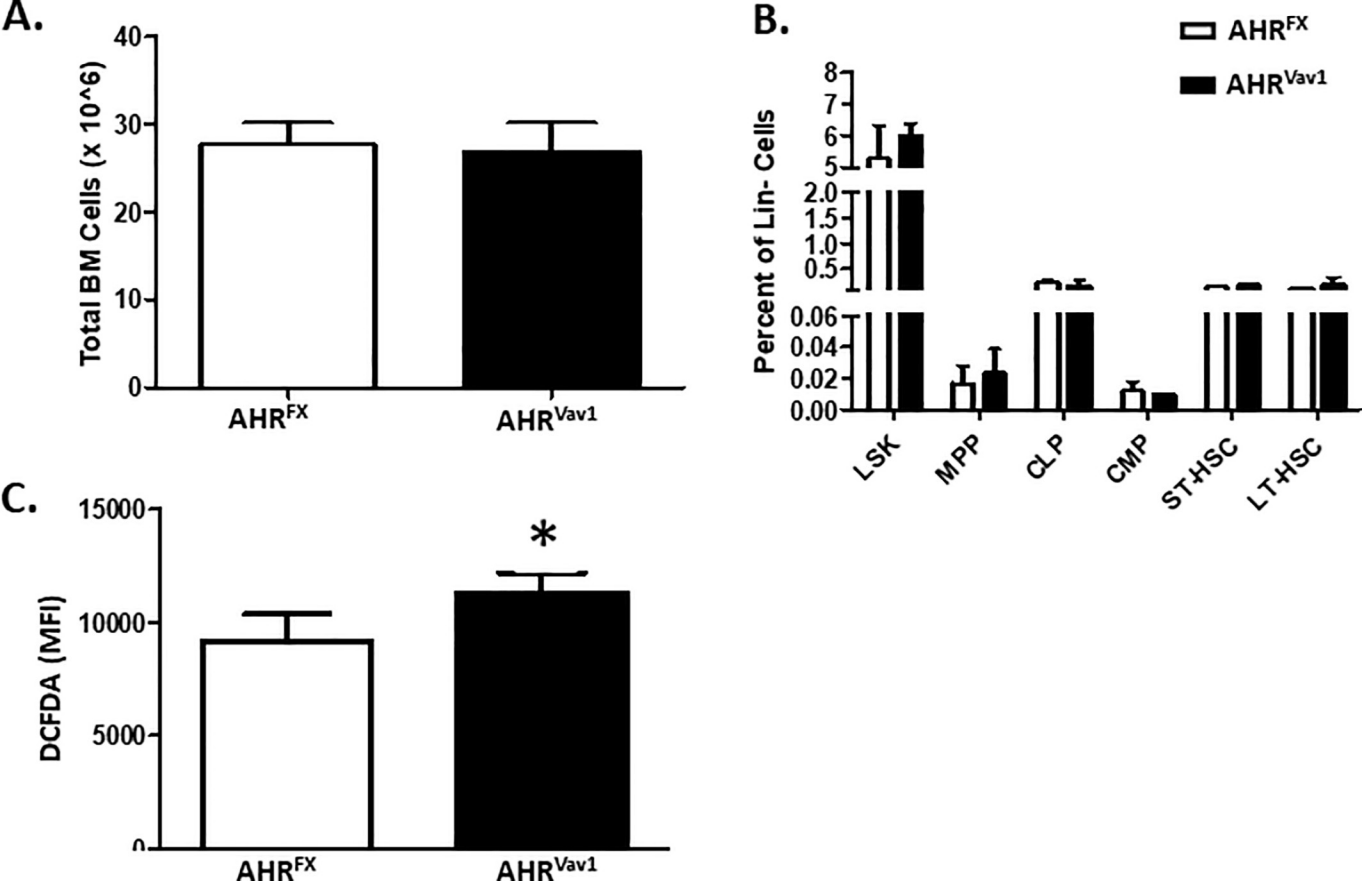
8-week old AHR^Vav1^ animals do not display alterations in total bone marrow cell counts or subpopulations and display heightened levels of ROS in hematopoietic LSK cells. (A). Total BM counts were determined for both AHR^Vav1^ and AHR^FX^ animals. (B) Flow cytometry was used to determine percentage of various hematopoietic subpopulations. N = at least 5 mice per group. (C) Flow cyometry was used to identify LSK cells, which had been co-stained with DCFDA to determine levels of oxidative species. Data presented as mean ± S.D. n = 5 per group. * Values significantly different from control (p<0.05).

Given that it was previously reported that global AHR-KO mice display alterations in BM subpopulations [6], we examined the frequencies of hematopoietic subpopulations in the BM of AHR^Vav1^ mice. In contrast to global AHR-KO mice, young AHR^Vav1^ mice display no significant alterations in the percentage of LSK, MPP, CLP, CMP, ST-HSC or LT-HSC in BM cells (Fig 3A and 3B). Given that AhR-KO mice also displayed decreased expression of ROS detoxifying enzymes such as Stra13 [7], we examined ROS levels in AHR^Vav1^ mice. LSK cells from AHR^Vav1^ mice had a small, but statistically significant, enhanced oxidative capacity as indicated by DCFDA staining (Fig 3C).

In order to determine if there are functional alterations in the hematopoietic system of young AHR^Vav1^ mice, we utilized the spleen colony forming assay [27] (S3 Fig). We observed no significant alterations in colony forming units at either 8 or 12 days following irradiation and injection of AHR^Vav1^ or AHR^FX^ cells. These data are consistent with the above data indicating no alterations in phenotype of BM from AHR^Vav1^ mice.

**Fig 4:**
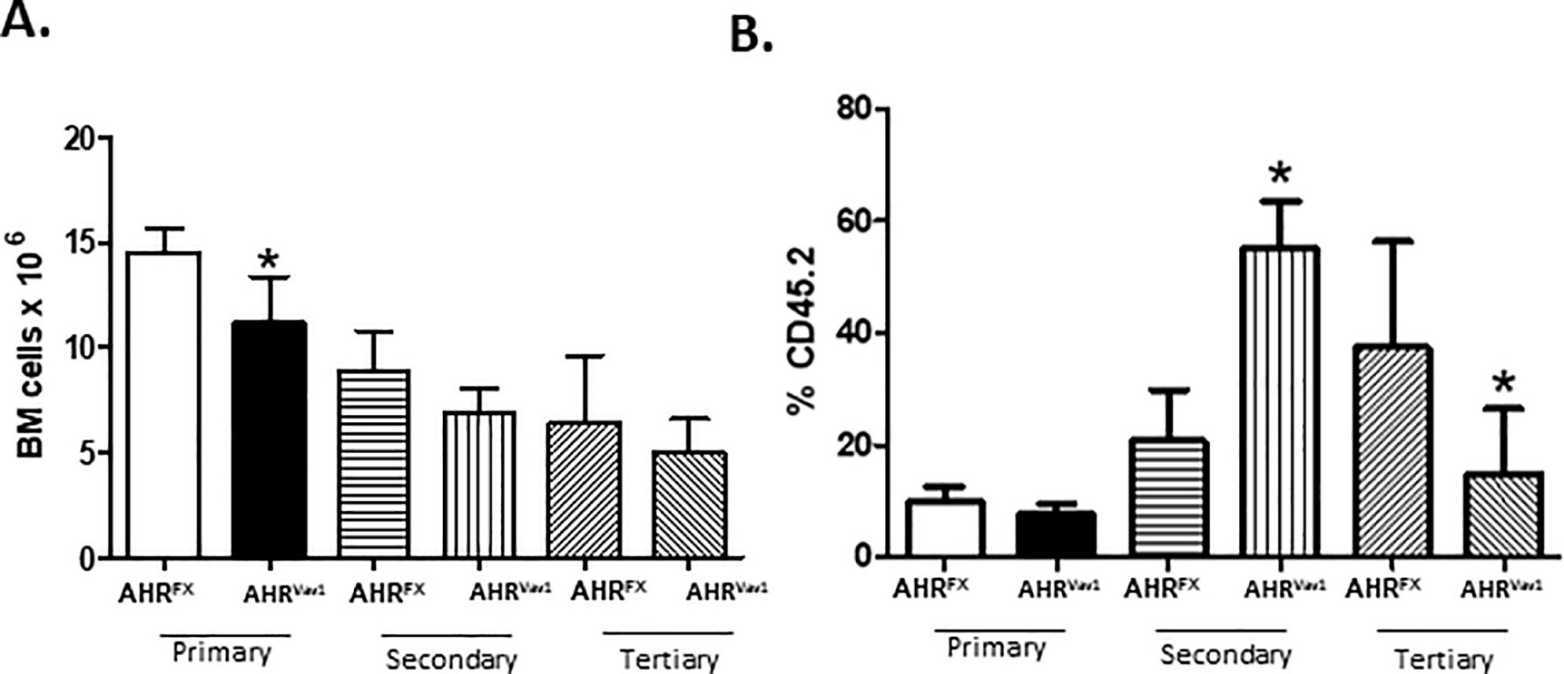
Cells from AHR^Vav1^ mice display a slight defect in serial transplantation. (A) Serial repopulation experiments were performed, and BM counts and (B) % CD45.2+ cell engraftment was determined at each successive stage. The extent of CD45.2+ cell engraftment was determined by flow cytometry. Data presented as mean ± S.D. n = 5 per group. * Values significantly different compared to AHR^FX^ donors as a control (p<0.05).

To determine whether conditional ablation of AHR from only the hematopoietic lineage affects LT-HSC function, we measured their ability to serially engraft and repopulate irradiated recipient mice. Serial transplantation forces LT-HSCs to exit quiescence and undergo regulated proliferation and differentiation, in order to repopulate blood lineages in the irradiated recipients. Thus, if conditionally ablating the AHR from only hematopoietic cells affects LT-HSC function, we would detect differences in cellular populations after serial transplantation. We observed a slight decrease in the total repopulation ability of cells from AHR^Vav1^ animals as indicated by the decreased number of CD45.2+ cells in primary recipients of AHR^Vav1^ cells (Fig 4A). However, AHR^Vav1^ cells appear to have enhanced proliferative potential at secondary transplant, indicated by the increase in the percentage of CD45.2+ cells in secondary recipients. This proliferative advantage did not persist to tertiary engraftment, as AHR^Vav1^ cells appear to exhaust prematurely when compared with AHR^FX^ cells at the same time point.

### AHR^Vav1^ mice have altered gene expression and signaling networks related to normal HSC function

**Fig 5:**
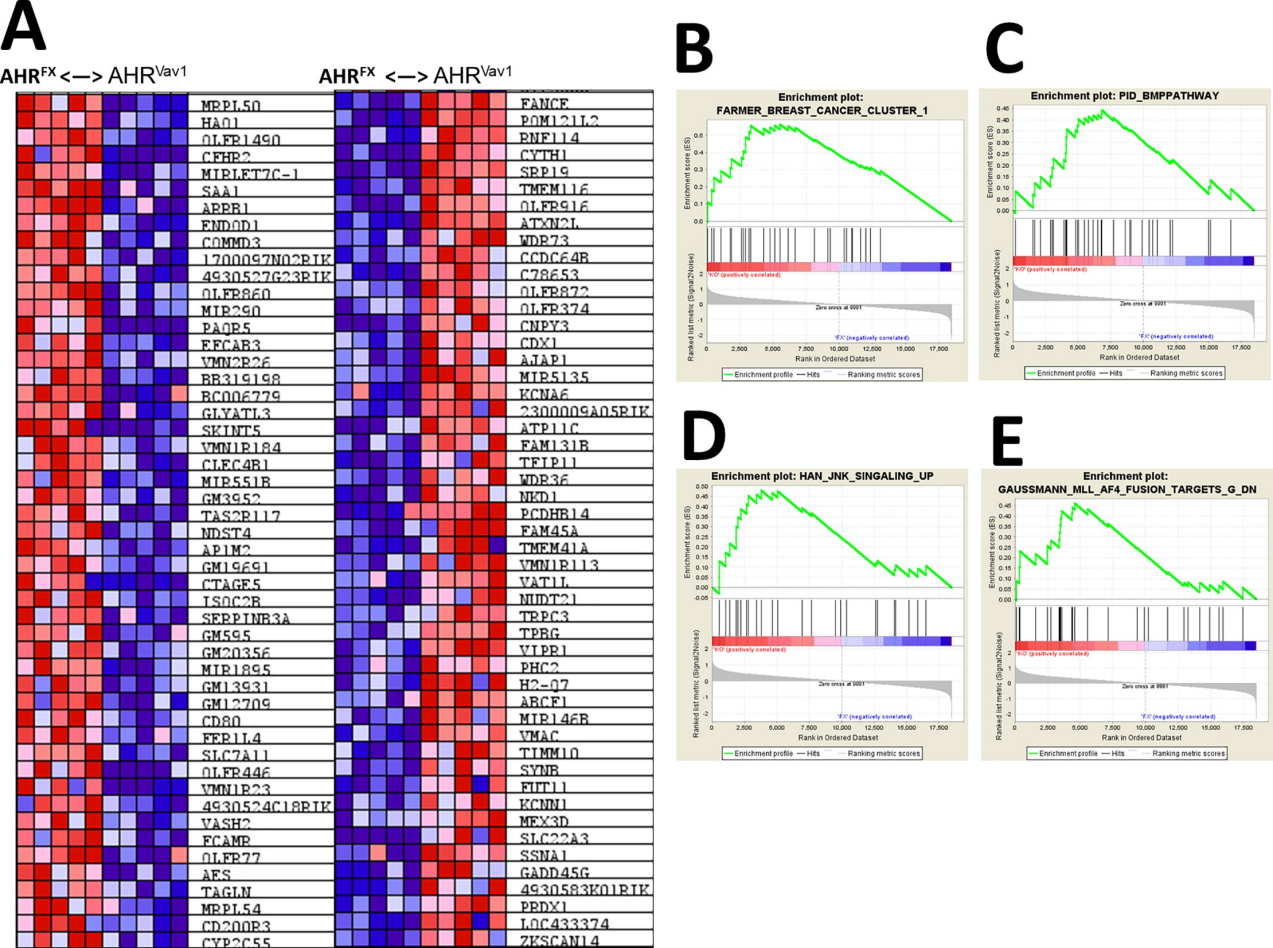
8 week old AHR^Vav1^ mice display altered gene expression in LT-HSCs: LT-HSCs from AHR^Fx^ and AHR^Vav1^ were sorted and analyzed by microarray. These analyses revealed differences in gene expression profiles between AHR^Fx^ and AHR^Vav1^ mice (A). These changes also indicate changes in specific pathways as revealed by Gene Set Enrichment Analyses (B—E).

**Fig 6:**
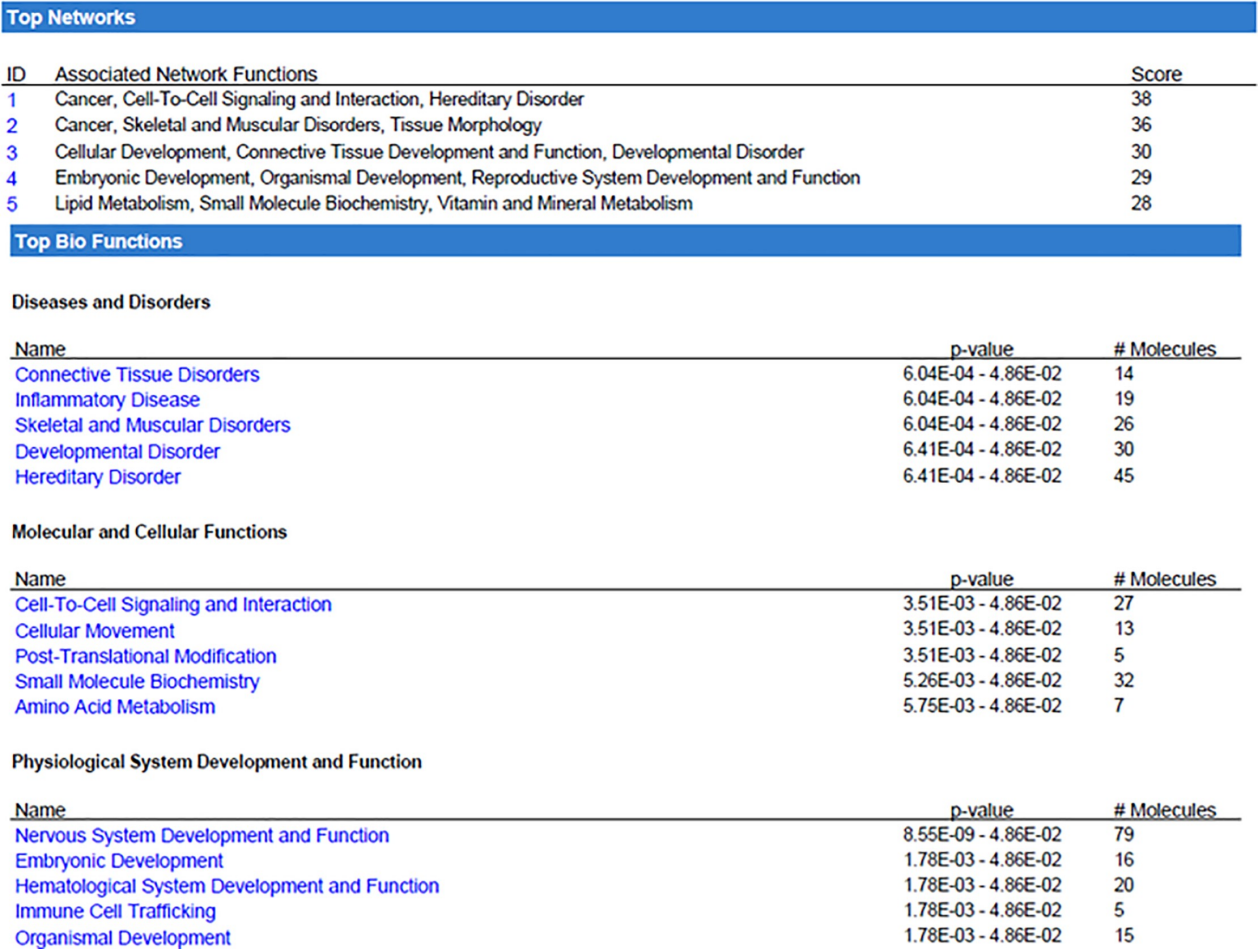
8-week old AHR^Vav1^ mice display alterations in signaling networks. Array data was analyzed using Ingenuity Pathway Analysis to determine which subcellular pathways and signaling networks might be altered with LT-HSCs due to lack of AHR expression within the hematopoietic compartment.

To determine the possible differential expression of genes due to lack of AHR, we analyzed sorted LT-HSCs from 2-month old female mice. As hypothesized, lack of AHR expression in hematopoietic cells is sufficient to drive differences in gene expression relative to AHR^FX^ controls (Fig 5A). Array data were also analyzed by Gene Set Enrichment Analysis (GSEA). Enrichment was determined in a variety of genes sets related to hematological disease or proliferative capacity (Fig 5B, 5C, 5D and 5E). To examine the potential biological impact of the genes determined to be altered by microarray, we next utilized Ingenuity Pathway Analysis (IPA). IPA indicated that there are likely alterations in AHR^Vav1^ LT-HSCs in pathways such as Cell-to-Cell Signaling and Interaction, Cellular Development, and Cancer (Fig 6). Consistent with the hypothesized role of AHR as a regulator of the hematopoietic system, the genes changed in LT-HSCs of 2-month old AHR^Vav1^ participate in hematological system development and function. Together, these proposed physiological functions generated by IPA are consistent with the hypothesis that lack of AHR is sufficient to induce alterations in gene expression in LT-HSCs, and that the induced alterations may play a role in the subsequent development of hematological disease or dysfunction. IPA was used to filter genes which change in LT-HSCs from both 8 week old AHR^Vav1^ and AHR-KO mice (AHR-KO microarray data from Gene Expression Omnibus database accession number: GSE 46976) and which also have putative AHR binding sites on their promoter (Supported by SABiosciences' proprietary database known as DECODE (DECipherment Of DNA Elements). These genes are presented in Table 2.

**Table 2:** Genes differentially regulated in HSCs from 2-month-old AHR-KO and AHR^Vav1^ mice, and which also contain AHR binding sites in their promoter region.

CKO vs FX	Fold Change	P	KO vs WT	Fold Change	P
Stra13	-4.52	0.02982	Stra13	-3.54	0.0014
Fam164c	1.83	0.01883	Fam164c	2.42	0.0434
Vmn1r218	2.58	0.04922	Vmn1r218	4.23	0.049
Fut11	-5.72	0.0216	Fut11	4.22	0.0193
Krtap7-1	2.84	0.01732	Krtap7-1	-3.07	0.0179
Cnpy3	-7.13	0.01154	Cnpy3	2.89	0.0154
Ankrd29	2.32	0.04337	Ankrd29	1.65	0.0497
Olfr1057	2.12	0.01515	Olfr1057	-2.81	0.0025
Olfr1058	-3.66	0.01532	Olfr1058	2.54	0.0147
Slc7a11	2.91	0.00689	Slc7a11	-42.12	0.0452
Cited4	1.51	0.03988	Cited4	-1.51	0.0244
Gm13040	1.54	0.04411	Gm13040	-3.23	0.0452
Pdp1	8.32	0.04899	Pdp1	5.42	0.0393
Arl9	2.55	0.02796	Arl9	-2.21	0.0233
Coro1c	2.52	0.03759	Coro1c	-2.96	0.0375
Gm19265	2.18	0.0448	Gm19265	-2.13	0.0003
C030030A07Rik	1.51	0.02305	C030030A07Rik	-1.96	0.0049
Wdr88	1.52	0.03527	Wdr88	-1.58	0.006
Vat1l	-1.92	0.00617	Vat1l	-2.14	0.0027
Olfr836	3.98	0.00839	Olfr836	3.95	0.0482
Cmc1	-2.41	0.01346	Cmc1	1.75	0.0141

**Fig 7:**
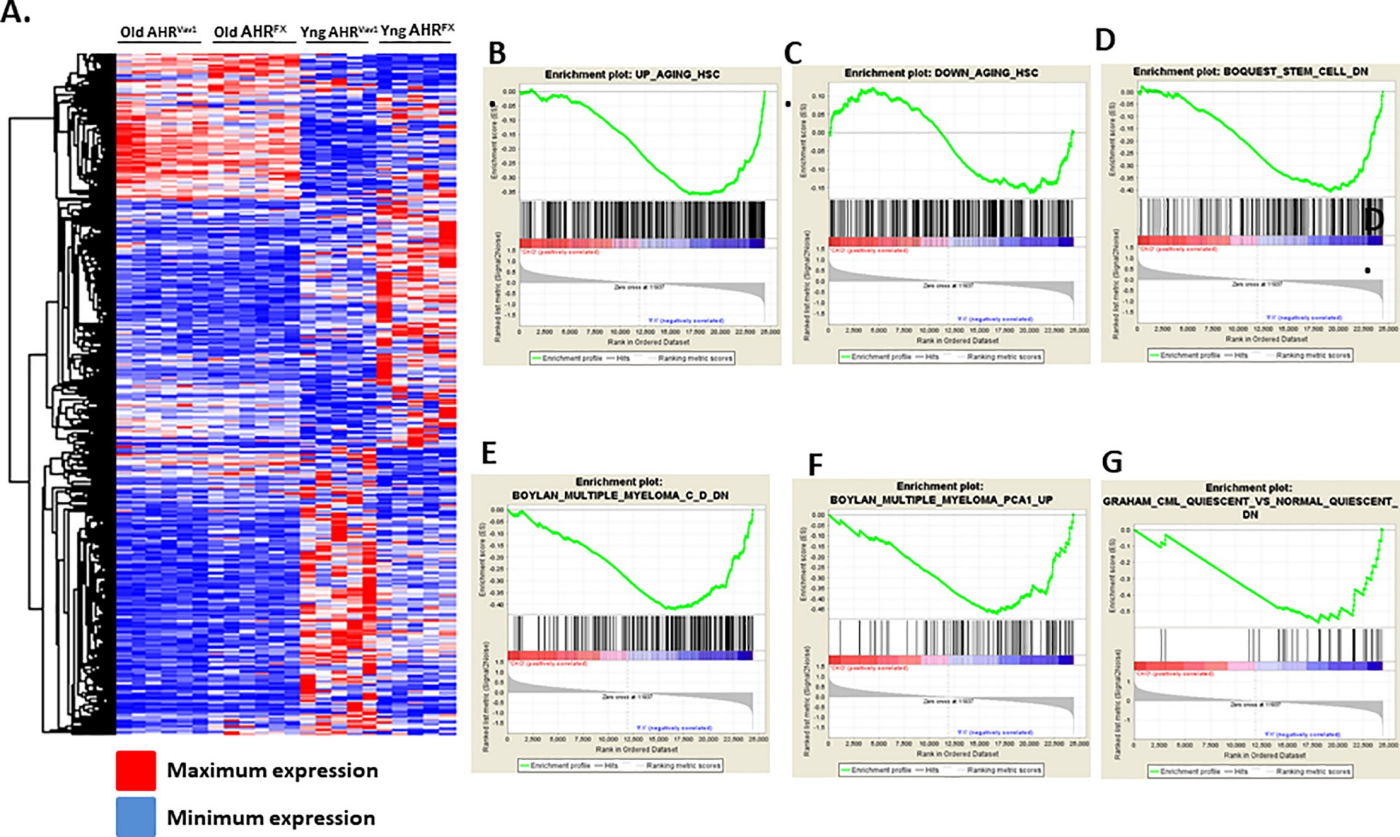
18-month old AHR^Vav1^ LT-HSCs display altered gene expression compared to AHR^FX^ controls. (A) Heatmap of array data from aging (Old) and young (Yng) AHR^FX^ and AHR^Vav1^ showing differential expression of genes. (B—G) Gene Set Enrichment Analysis was performed on array data from LT-HSCs from 18-month old AHR^FX^ and AHR^Vav1^ mice. Gene profiles are altered in AHR^Vav1^ mice.

**Fig 8:**
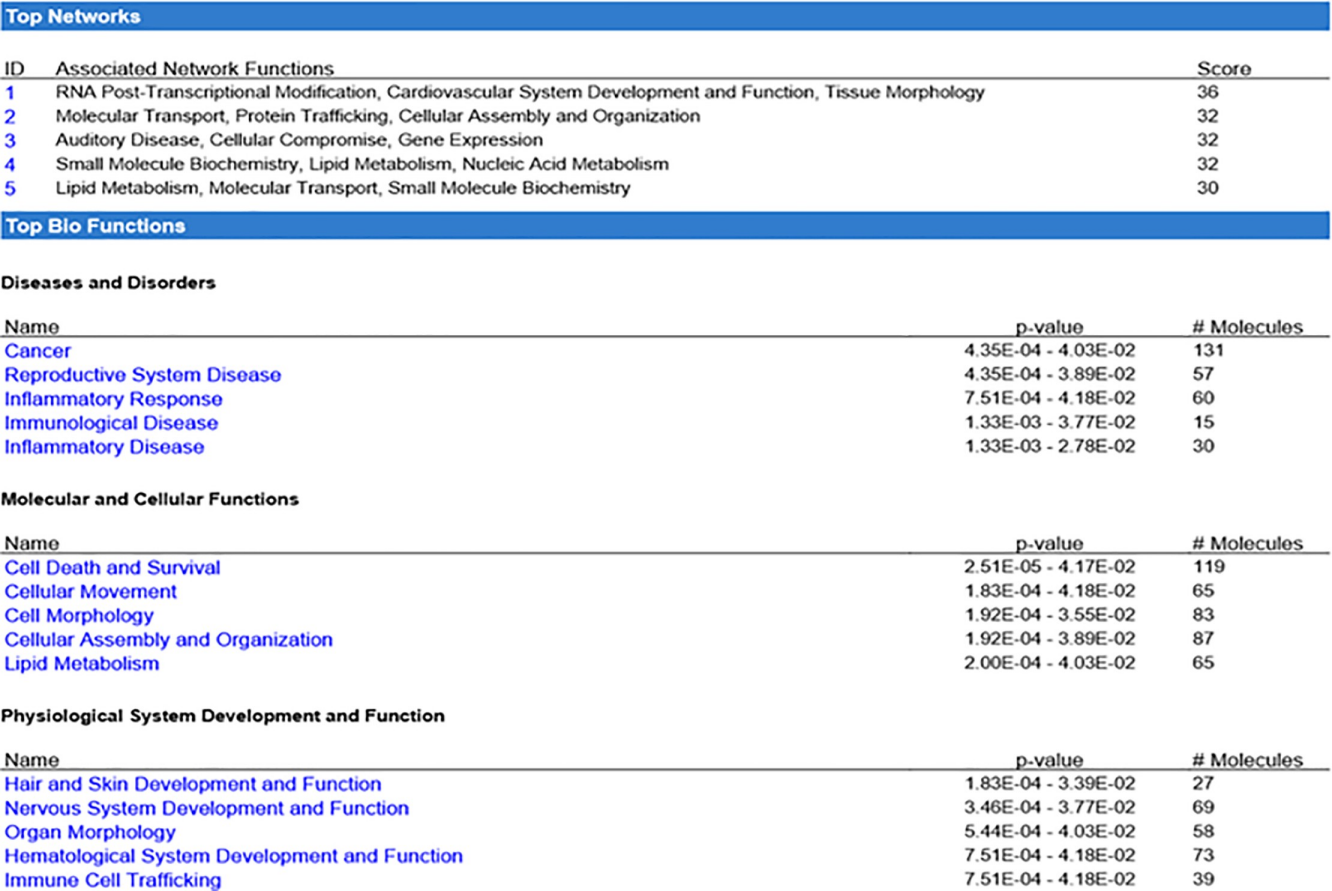
Ingenuity Pathway Analysis of differentially expressed genes in LT-HSCs from 18-month-old AHR^Vav1^ mice. Array data was uploaded to IPA, which was set to include to include only observations that have been experimentally observed, using direct and indirect relationships.

To examine the potential functional impact of lack of AHR in the hematopoietic system throughout aging, we repeated the array and GSEA/pathway analyses in 18-month old AHR^Vav1^ mice. These analyses indicated that LT-HSCs from old AHR^Vav1^ mice have differential gene expression compared to floxed controls (Fig 7A). Array data were also analyzed by GSEA. Enrichment was determined in a variety of genes sets related to hematological disease or proliferative capacity and HSCs aging (Fig 7B–7G). IPA analysis indicated that there are alterations in AHR^Vav1^ LT-HSCs in genes functionally significant for hematopoietic stem cell function or activity (Fig 8). A different subset of altered genes was examined which possess putative AHR binding sites in aged AHR^Vav1^ and AHR-KO (AHR-KO microarray data from Gene Expression Omnibus database accession number: GSE 67378) are presented in Table 3. Ingenuity Pathway Analysiswas used to filter this list, and which also have putative AHR binding sites confirmed using SA Biosciences website. These data are presented Table 3.

**Table 3:** Overlapping genes differentially regulated in HSCs from both 18-month-old AHR-KO and AHR^Vav1^ mice.

Up-regulated genes in 18-month-old AHR-KO and AHR^Vav1^	Down-regulated genes up-regulated genes in 18-month-old AHR-KO and AHR^Vav1^
1700030F18Rik	1700003H04Rik
A630023A22Rik	1700100L14Rik
AGBL4	2610305D13Rik (includes others)
ANAPC1/ANAPC1P1	4930459I23Rik
BIRC6	AP4S1
EBF3	ARAP1
FOXB1	C16orf93
GINS1	CYP2D6
Gm4836	Gm19404 (includes others)
KRTAP2-3 (includes others)	IL1RAP
MBIP	Lce1a2
MRPS31	MAGEL2
Mup1 (includes others)	Mir3087
Olfr887	PERP
PSMG3	RUNX1T1
RPS15A	SEMA3C
SF3B4	Speer4a (includes others)
Trhr2	Ssty1 (includes others)
Zfp932 (includes others)	TMEM159
	Vmn1r188 (includes others)
	Vmn2r66 (includes others)
	Zscan4b (includes others)

**Fig 9:**
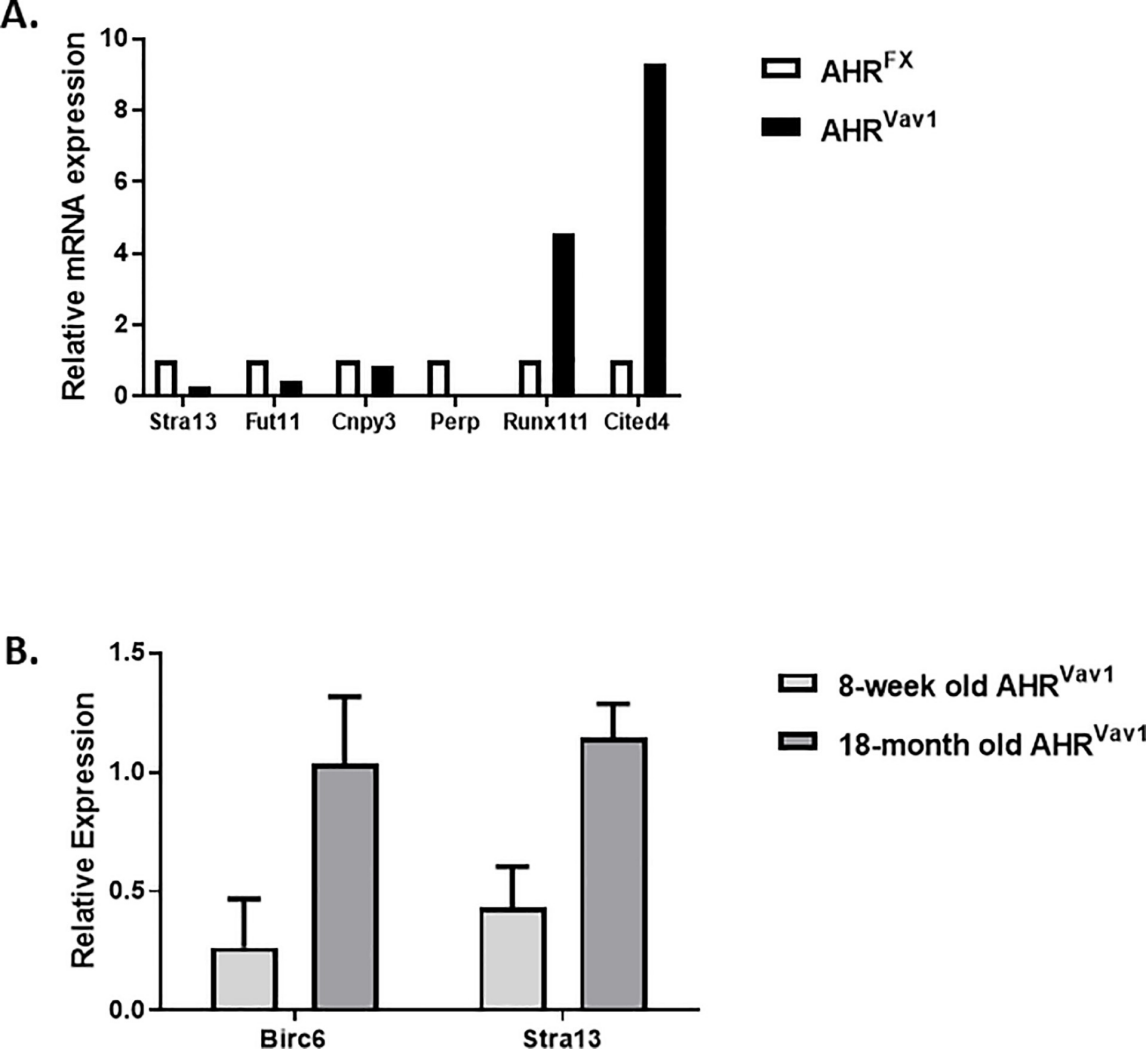
LT-HSCs from AHR^Vav1^ mice have altered expression of genes directly involved in hematopoiesis (A) LT-HSC samples were sorted from BM cells and analyzed for selected genes proposed to have a role in HSC. Samples are from 6-7-week old animals. (B) Samples are from 6-8-week old female animals for the young comparison, 18-month old female mice for the old comparison. Each gene is normalized relative to GAPDH expression in the AHR^FX^ animals at the indicated age. Data shown as mean +/- SD for at least three replicates. * = P<0.05.

To confirm that certain genes identified utilizing microarray and bioinformatics approaches are truly differentially expressed in AHR^Vav1^ mice, we chose to examine genes linked to hematopoietic development. We used qPCR to examine the expression of genes related to hematopoiesis at both ages, which included Birc6 and Stra13 (Fig 9A and 9B). Consistent with the hypothesis that AHR may regulate these genes, and hematopoietic aging in a broad sense, we observed altered relative expression of both Stra13 and Birc6 in LT-HSCs for both ages of AHR ^Vav1^ animals.

## Discussion

The data presented here are consistent with the hypothesized role of AHR as a potential intrinsic regulator of genes in HSCs. In consideration of data previously reported for AHR-KO mice, where we observed altered functional capacity and abnormal proliferation [6], they also suggest that AHR signaling in non-hematopoietic “niche” tissue assists in the regulation of HSCs, and the absence of this signaling in AHR-null allele mice is partially responsible for producing the phenotypes observed.

Interestingly, AHR^Vav1^ mice do appear to phenocopy enhanced oxidative capacity of LSK cells, and this finding is consistent with a prior report in AHR-KO mice [7] and with the described crosstalk between AHR and the anti-oxidative transcription factor Nrf2. [28]. Functional assays of progenitor and HSC function indicate that AHR^Vav1^ mice are not altered at the level of progenitors as indicated by spleen colony forming units. This finding is consistent with the lack of alterations observed in BM progenitors assessed by flow cytometry. However, AHR^Vav1^ mice do display alterations in serial repopulation assays that resemble expansion and early exhaustion at the secondary and tertiary transplant analyses, respectively. While surprising that a defect only became apparent at the secondary stage of engraftment, these data are is consistent with AHR regulating at least some of the aspects of engraftment in BM, and with a previous report that activation of AHR in HSCs alters gene expression related to migration and trafficking [3].

The lack of phenocopy between AHR-KO mice and AHR^Vav1^ mice can potentially be due to the presence of AHR in non-hematopoietic cells in the AHR^Vav1^ mice. In addition, the young age of the mice examined could potentially play a role in the absence of the AHR-KO phenotype. Aging is a significant risk factor for hematological diseases [29], and dysfunction of hematopoietic system [30–32]. It is possible that the AHR^Vav1^ mice could develop a phenotype with aging that is more consistent with observed phenotypes in AHR-KO mice such as splenomegaly, white cell expansion, or LSK expansion [6, 7]. Future studies will address this issue. However, notably there were significant deficits observed following the serial transplant of HSCs from young animals. Serial transplantation mimics some aspects of HSC aging. The observation that a defect becomes apparent in the secondary stage of engraftment with AHR^Vav1^ mice as donors is consistent with the hypothesis that AHR promotes long term fitness of hematopoietic stem cells. Serial repopulation mimics proliferative stress and aging, and it is intriguing that a defect became apparent at later iterations of engraftment. This is suggestive that expression of AHR is required for long term HSC maintenance and function.

In order to determine what gene expression changes could potentially contribute to HSC dysfunction, we examined gene expression profiles in LT-HSCs from AHR^Vav1^ mice. Gene expression profiles in LT-HSCs from young AHR^Vav1^ mice are substantially altered. Hierarchical clustering of the genes reveals alterations in genes such as FANCE, which is a critical component of DNA repair machinery in HSCs [33], and CFHR2 which may have regulatory roles for immune cells and complement activation [34]. Gene Set Enrichment Analysis indicates enrichment for genes in hematopoietic and cancer related gene sets from the Molecular Signatures Database. These genes include Jun and Fos, which are important regulators of HSC function [35, 36]. The genes altered examined fall into gene sets and pathways that are likely to have functional impact on hematopoietic stem cell function or output.

Ingenuity Pathway Analysis indicated that the genes differentially expressed in AHR^Vav1^ mice are related to cell networks such as Cancer, Cell-to-Cell Signaling and Interaction, and Cellular Development. This finding is consistent with known and hypothesized roles for AHR in a variety of cell types [37–39]. The top biological functions predicted by IPA include Cellular Movement, Amino Acid Metabolism, Immune Cell Trafficking, Small Molecule Biochemistry and Hematological System Development and Function. These proposed networks are again consistent with known and hypothesized roles for AHR [3], and with the proposed activation of AHR by a variety of endogenous ligands [40–43]. qPCR confirmation of a subset of genes significant to the hematopoietic system revealed that at both young and old ages, AHR^Vav1^ mice have differential gene expression. Stra13 is involved in the detoxification of reactive oxygen species. We have previously identified Stra13 as differentially regulated in HSCs lacking AHR using a global deletion mouse (AHR-KO) model [7]. This finding suggests that AHR is an important regulator of genes which control ROS detoxification, as Stra13 has been decreased in both AhR-KO and AhR ^Vav1^ mice. Birc6 is a regulator of apoptosis and has been described as being deregulated in acute myeloid leukemia [44]. Interestingly, knockdown of Birc6 in cell lines used to model promyelytic leukemia impairs neutrophil differentiation, and AhR regulation of Birc6 is suggested by our data. This finding is consistent with the hypothesis that AHR is a critical regulator of gene expression in LT-HSCs. Many of the genes we observed to be changed are differentially altered (directionality) between the two types of mouse model used to study AhR (globally deficient, vs HSC deficient). This lack of phenocopy is consistent with our hypothesis that non-hematopoietic effects are driving some of the changes in global AHR-KO animals, and further highlights the utility of using AHR^Vav1^ mice to identify genes directly regulated by AHR in hematopoietic cells.

Our lab has recently published work which suggests that AHR-KO mice have changes in the cellular composition of BM stromal cells [45]. We also observed higher BM cell counts in chimeric mice having WT hematopoietic cells transplanted into AHR-KO host. This study suggests that lack of AHR within BM stromal cells has a role in determining HSC phenotype and function in AHR-KO mice. HSCs from AHR-KO mice have reduced expression of Angiopoietin 1 (Angpt1). It has been reported that changes in Angpt1 gene expression in HSCs may induce secondary changes in BM niche [46]. Angpt1 is also expressed by niche osteoblast cells, which promotes quiescent HSCs in BM niche [47]. The observed phenotypic differences between AHR-KO and AHR^Vav1^ HSCs may be not only due to *AHR* cell-intrinsic regulation of HSCs, but an extrinsic effect of an AHR-regulated BM niche environment that may play a role in HSC homeostasis and function.

Although we did not observe the same phenotype, function and gene profiles of AHR^Vav1^ HSCs as in AHR-KO mice, common changes in several genes were observed. The expression of *pdp1* (up-regulated) and *stra13* (down-regulated) were altered in both global and conditional AhR KO models. These genes are associated with aging and longevity, as well as oxidative stress [48,49]. When these mice age they have several common genes up-regulated (*agbl4*, *birc6*, *anapc1*) and down-regulated (*runx1t1*, *perp*); these play a role in megakaryopoiesis, apoptosis, cell cycle and aging related changes, apoptosis and leukemia [45, 50, 51].

Notably, *Vav-1*, and, in this model, *Cre*-recombinase, are expressed in hematopoietic cells at about mouse gestational day 11 [21, 26]. As such, it is possible that despite the multiple gene changes in HSCs from AHR^Vav1^ mice, some compensatory gene and functional changes occurring (in HSCs and/or BM niche cells) during the lifetime of the AHR^Vav1^ animals could contribute to the lack of complete phenocopy compared to AHR-KO mice. It is also possible that some AHR-dependent pre-programmed event taking place prior to gestational day 11 could “lock in” some aspects of HSC regulation. Other studies, such as the examination of an inducible KO model, may address some of these issues.

In conclusion, the data presented here suggest that lack of AHR in HSCs is sufficient to induce alterations in HSC gene expression, but these gene changes do not appear to induce significant alterations in HSC output/function at 2-months of age, other than in the context of serial transplantation, that were observed in AHR-KO mice. These findings indicate the need to examine the role of AHR signaling in non-hematopoietic stromal cells of the BM (or more distant tissues), and how such signaling can ultimately influence the function or activity of HSCs. The enhanced levels of oxidative species in both AHR^Vav1^ and AHR-KO mice are supportive of a role for AHR:Nrf2 crosstalk in maintaining HSC oxidative levels and may represent an important protective mechanism in HSC biology. These data also suggest the need to examine aging of AHR^Vav1^ mice to determine the impact that deficiency in AhR may have on the development of hematological disease.

## Supporting information

S1 Fig Organ weights are not altered in 8 week old AHR^Vav1^ mice compared to AHR^FX^ controls. Organs were collected and wet mass was determined. No significant differences were detected.
(TIF)

S2 Fig 8 week old AHR^Vav1^ mice do not have altered splenic cell counts or subpopulations compared to AHR^FX^ controls. (A) Cell counts for a single spleen of the indicated genotypes. (B) Flow cytometric analysis of splenic cell subpopulations.
(TIF)

S3 Fig 8 week old AHR^Vav1^ HSCs trend towards enhanced spleen colony foming ability. Spleen colony forming assays were performed as described, and colonies counted at 8 and 12 days. Slightly higher but non-significant differences were observed.
(TIF)
