# Supplementary figures and images for: Conditional deletion of Ahr alters gene expression profiles in hematopoietic stem cells
Source: PLoS One. 2018 Nov 2;13(11):e0206407. doi: 10.1371/journal.pone.0206407 (PMC6214519; doi:10.1371/journal.pone.0206407)

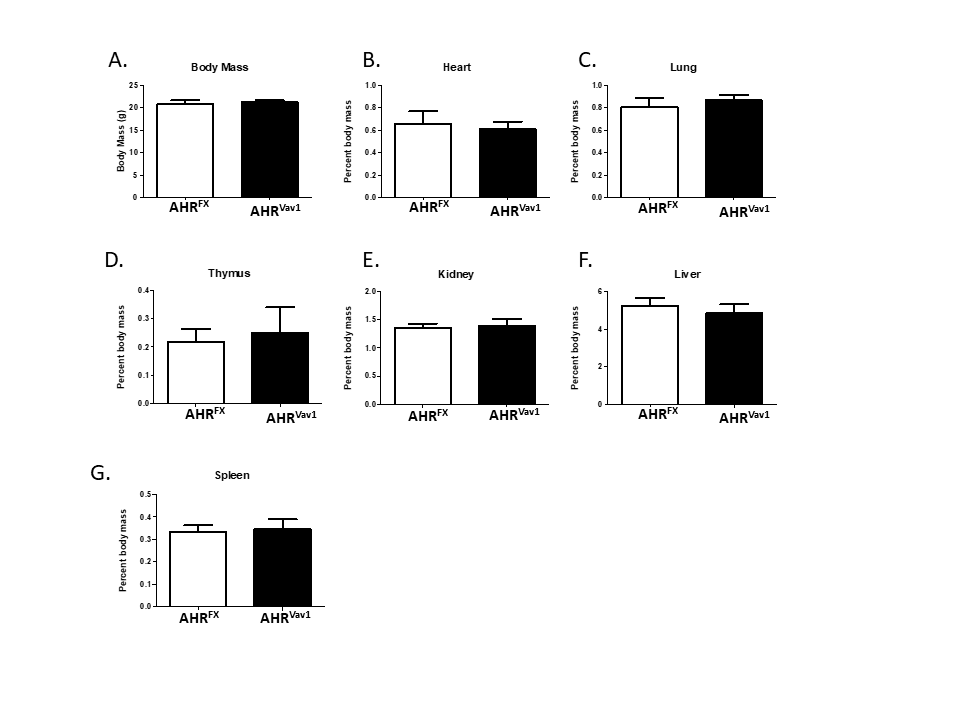

Supplement: S1 Fig — Organs were collected and wet mass was determined. No significant differences were detected. (TIF) [file pone.0206407.s001.tif]

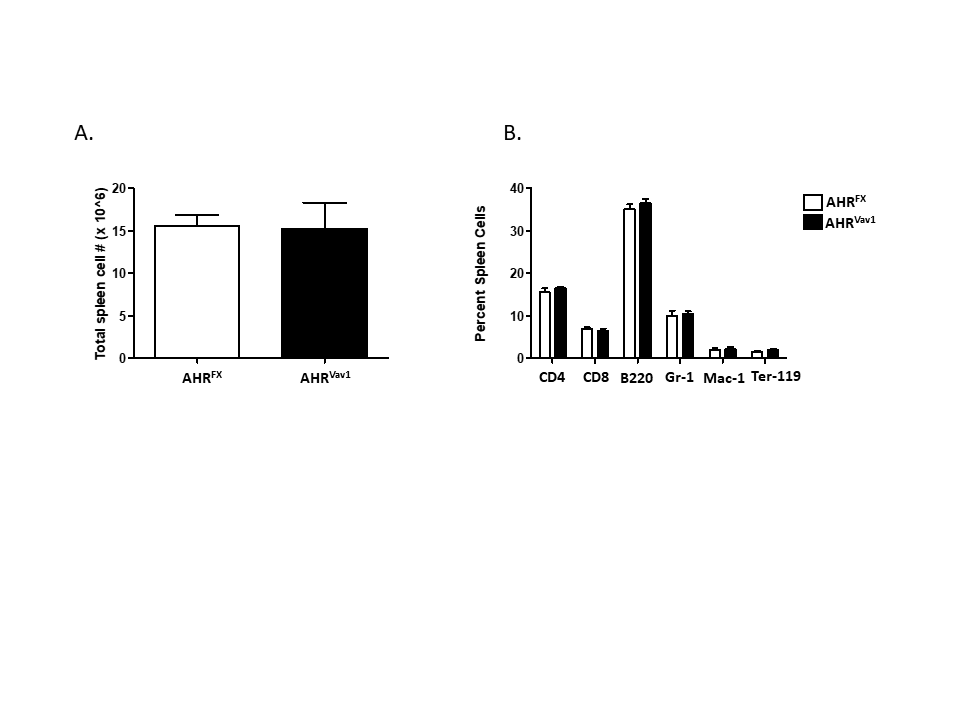

Supplement: S2 Fig — (A) Cell counts for a single spleen of the indicated genotypes. (B) Flow cytometric analysis of splenic cell subpopulations. (TIF) [file pone.0206407.s002.tif]

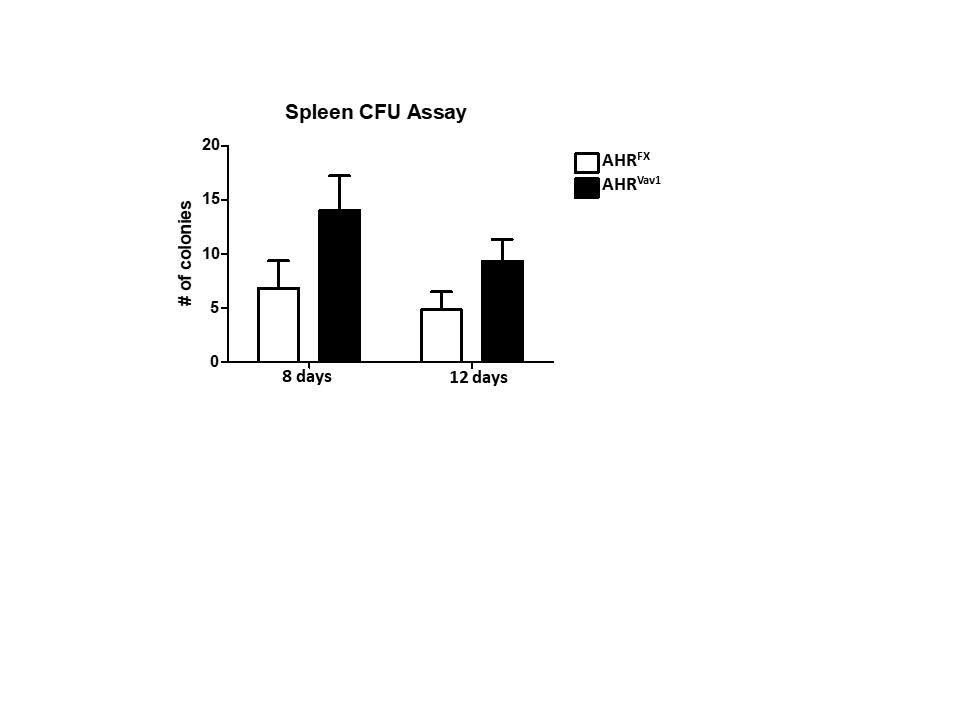

Supplement: S3 Fig — Spleen colony forming assays were performed as described, and colonies counted at 8 and 12 days. Slightly higher but non-significant differences were observed. (TIF) [file pone.0206407.s003.tif]
